# Self perception and facial emotion perception of others in anorexia nervosa

**DOI:** 10.3389/fpsyg.2015.01181

**Published:** 2015-08-10

**Authors:** Andrea Phillipou, Larry A. Abel, David J. Castle, Matthew E. Hughes, Caroline Gurvich, Richard G. Nibbs, Susan L. Rossell

**Affiliations:** ^1^Department of Optometry and Vision Sciences, The University of MelbourneMelbourne, VIC, Australia; ^2^Department of Psychiatry, The University of MelbourneMelbourne, VIC, Australia; ^3^Department of Mental Health, St Vincent’s Hospital, MelbourneVIC, Australia; ^4^Department of Psychiatry, St Vincent’s Hospital, MelbourneVIC, Australia; ^5^Faculty of Health Sciences, Australian Catholic University, MelbourneVIC, Australia; ^6^Brain and Psychological Sciences Research Centre, Swinburne University of Technology, MelbourneVIC, Australia; ^7^Monash Alfred Psychiatry Research Centre, MelbourneVIC, Australia

**Keywords:** eating disorders, fMRI, eyetracking, eye movements, affect

## Abstract

**Background:** Whether individuals with anorexia nervosa (AN) are able to accurately perceive emotions from faces of others is unclear. Furthermore, whether individuals with AN process images of their own face differently to healthy individuals has thus far not been investigated. Therefore, the aim of this study was to investigate facial affect processing and the processing of one’s own face through measures of emotion identification, functional magnetic resonance imaging (fMRI) and eyetracking.

**Methods:** Twenty-four females with AN and 25 matched healthy control participants were presented with an implicit emotion processing task during fMRI and eyetracking, followed by an explicit emotion identification task.

**Results:** The AN group were found to ‘hyperscan’ stimuli and avoided visually attending to salient features of their own face images. Results of the fMRI revealed increased activity to own face stimuli in AN in the right inferior and middle temporal gyri, and right lingual gyrus. AN participants were not found to display emotion identification deficits to the standard emotional face stimuli.

**Discussion:** The findings are discussed in terms of increased anxiety to disorder-relevant stimuli in AN. Potential clinical implications are discussed in relation to the use of eyetracking techniques to improve the perception of self in AN.

## Introduction

Anorexia nervosa (AN) is a psychiatric condition characterized by a significantly low body weight, a fear of weight gain and a disturbance in the experience of one’s own body weight or shape (American Psychiatric Association, 2013). A common pathognomonic psychological factor in AN is the disturbance of body image (Tovée et al., 2000). AN also significantly overlaps with anxiety disorders in terms of symptoms and phenomenology, particularly obsessive compulsive disorder (Thiel et al., 1995; Kaye et al., 2004). Additionally, AN has long been associated with deficits in the perception of emotion; in her pioneering work, Bruch (1962) observed a marked deficiency in the description of feelings and emotional responses in patients with AN. Later research has linked these deficiencies to the construct of alexithymia, which is defined as a difficulty in identifying and describing subjective feelings, a difficulty in distinguishing between feelings and the bodily sensations of emotional arousal, an externally oriented cognitive style, and a lack of imaginal capacity and fantasy (Nemiah et al., 1976). Increased rates of alexithymia have been consistently reported in AN (Bourke et al., 1992; Kessler et al., 2006; Troop et al., 2006), though whether individuals with AN have difficulty in the processing of other people’s emotions has not been significantly elucidated.

A number of studies have reported that AN patients perform poorly on tasks probing human face emotion identification (also referred to as ‘facial affect processing’). Some authors have reported that this deficit was not specific to any particular emotion (Jänsch et al., 2009), while others have found deficits that are specific to the identification of surprise (Kessler et al., 2006; Legenbauer et al., 2008), sadness (Kucharska-Pietura et al., 2004; Pollatos et al., 2008), disgust (Pollatos et al., 2008), and fear (Kucharska-Pietura et al., 2004). In only one study of which we are aware, the authors did not find any emotion identification deficit in AN (Mendlewicz et al., 2005). The consistency of these findings has prompted researchers to explore the neurobiological basis of this impairment in AN using neuroimaging techniques.

To date, only two published functional magnetic resonance imaging (fMRI) studies have investigated facial affect processing in AN. Fonville et al. (2014) found that AN patients exhibited increased blood-oxygen level dependent (BOLD) activation in the right fusiform gyrus in response to mildly happy, prototypically happy, and neutral human face expressions compared to controls. In contrast, Cowdrey et al. (2012) did not find any group differences with their paradigm that used fearful and happy human face expressions. However, the findings of these studies have limited utility as neither contrasted the affect images with neutral affect images to control for differential between group face processing *per se*; Fonville et al. (2014) contrasted the emotions to a baseline (fixation cross) and Cowdrey et al. (2012) contrasted fearful and happy conditions with one another.

Another related set of studies have analyzed visual scanpaths and have revealed critical information for understanding face emotion processing in a range of psychiatric conditions. During face stimulus processing, healthy individuals focus on salient features such as the eyes, nose, and mouth (Walker-Smith et al., 1977), whereas patients with psychiatric conditions such as schizophrenia and autism spectrum disorder, show reduced visual attention to these features (Loughland et al., 2002; De Wit et al., 2008). Furthermore, although poorer attention to salient features has been found in psychiatric populations such as schizophrenia during implicit emotion processing tasks, typical attention to salient features has been found when explicit task instructions are given (Delerue et al., 2010). Individuals with psychiatric conditions also show different scanpath strategies: those with anxiety disorders such as social anxiety disorder ‘hyperscan’ (increased scanpath lengths with fixations of shorter duration) face stimuli (Horley et al., 2003), whereas those with schizophrenia show a restricted scanpath of fewer fixations of longer duration, and reduced scanpath lengths (Loughland et al., 2002). Whether individuals with AN also exhibit discrepant visual scanpath behavior when viewing face stimuli relative to healthy individuals, has thus far not been thoroughly investigated. During a free-viewing task where participants were presented with whole body stimuli and face stimuli, Watson et al. (2010) reported reduced attention to the eye region of face stimuli, and less time visually attending face regions when whole bodies were presented in AN. However, the findings of that study are limited as no attempt was made to standardize the stimuli acquired from a dating website. Freeman et al. (1991), on the other hand, presented participants images of their own bodies photographed in a black leotard and reported that while healthy controls spent relatively the same amount of time focusing on four interest areas (face, chest, abdomen, and legs), individuals with AN spent more time looking at the their legs and abdomen and less time looking at the face, suggesting an avoidance of fixating one’s own face in AN.

The aim of the current study was to investigate the processing of emotional faces of others, and faces of self, in AN. Participants performed an implicit emotion processing task that involved gender identification of stimuli while undergoing fMRI and eyetracking, and an explicit emotion identification task outside the scanner during eyetracking. Functional imaging studies of emotional face perception are typically performed as implicit tasks, such as gender decision tasks, rather than explicit tasks as they are less cognitively demanding and do not interfere with emotional processing. As explicit tasks have a higher cognitive demand, more frontal areas are involved and it is more difficult to observe the areas involved in emotion processing (Critchley et al., 2000; Gorno-Tempini et al., 2001). Performing the task both implicitly and explicitly also allowed for the investigation of emotion perception and scanpaths under different conditions. As scanning behaviors have not previously been specifically investigated in AN, individuals with AN were expected to exhibit similar scanning behaviors to related conditions such as anxiety disorders, namely, hyperscanning of face stimuli during both tasks (i.e., increased fixations of shorter duration). Similarly to other psychiatric conditions, we further hypothesized that AN participants would show an avoidance of salient features of the emotional face stimuli during the implicit task. As typical attention to salient features has been found when explicit task instructions are given in populations that demonstrate poor attention to salient features during implicit tasks (Delerue et al., 2010), groups were not expected to differ in areas of attentional focus during the explicit task. In relation to participants’ own face stimuli and given the avoidance of looking at one’s own face as reported by Freeman et al. (1991), the AN group were hypothesized to show less attention to salient features during both tasks. Individuals with AN were also expected to show emotion identification deficits to face stimuli displaying negative emotion, but were not expected to show emotion recognition difficulties to their own face when they were asked to display a neutral expression. Related to this hypothesis, the AN group were expected to manifest reduced activity in limbic areas of the brain to negative affect stimuli, relative to neutral control faces; and to show reduced activity to stimuli of their own face in frontoparietal brain areas which are involved in the processing of ones’ own face (Uddin et al., 2005).

## Materials and Methods

This study was approved by the human research ethics departments at The University of Melbourne, Swinburne University of Technology, The Melbourne Clinic, The Austin Hospital, and St Vincent’s Hospital; all in Melbourne, VIC, Australia. Informed written consent was obtained from all participants. All procedures contributing to this work comply with the ethical standards of the relevant national and institutional committees on human experimentation and with the Helsinki Declaration of 1975, as revised in 2008.

### Participants

Twenty-four right-handed individuals with AN and 25 healthy control (HC) individuals were recruited for the study. One participant in the AN group declined to have her own face included among the stimuli and her data were not included in the analysis. A programming error resulted in one HC participant being presented with too few own face stimuli and her data were excluded. Technical difficulties encountered with use of the eyetracking equipment in the MRI resulted in the data of three AN participants and three HC participants being excluded, allowing eyetracking analyses to be conducted on 20 AN and 21 HC participants. All 23 AN and 24 HC participants’ data were included in the fMRI and emotion identification analyses.

HCs were recruited through public advertisements, whereas AN participants were recruited through public advertisements, the Body Image and Eating Disorders Treatment and Recovery Service at the Austin and St Vincent’s Hospitals, and The Melbourne Clinic (all located in Melbourne, VIC, Australia). All participants were English speaking, had no history of significant brain injury or neurological condition, no significant ocular pathology and normal (or corrected to normal) visual acuity. Controls were required to have no history of an eating disorder or other mental illness; they were also required to not be taking any medications apart from hormonal contraceptives (11 HC participants were taking this medication). AN participants were instructed to continue with their normal medications, which were: selective serotonin reuptake inhibitors (SSRIs) (10), atypical antipsychotics (10), benzodiazepines (5), serotonin-noradrenaline reuptake inhibitors (SNRIs) (3), hormonal contraceptives (3), melatonergic antidepressants (2), noradrenergic and specific serotonergic antidepressant (NaSSA) (1), and cyclopyrrolones (1). Medications that patients were taking, such as benzodiazepines and atypical antipsychotics have been found to moderately reduce saccadic peak velocity (Reilly et al., 2008), but do not affect scanpaths to the best of our knowledge.

The Mini International Neuropsychiatric Interview, 5.0.0 (MINI; Sheehan et al., 1998) was used to screen participants for major Axis I psychiatric disorders according to the Diagnostic and Statistical Manual of Mental Disorders (DSM-IV). It was also used to confirm diagnoses of AN, with the exception of the amenorrhea criterion which is not included in DSM-5 criteria. AN was required to be the primary diagnosis of the AN group; participants with comorbid psychiatric conditions, other than psychotic conditions, were not excluded as this would not have represented a typical AN sample.

Premorbid intelligence was estimated using the Wechsler Test of Adult Reading (WTAR; Wechsler, 2001). Eating disorder symptomatology was investigated with the Eating Disorders Examination Questionnaire (EDE-Q; Fairburn, 2008) and alexithymia with the Toronto Alexithymia Scale (TAS-20; Bagby et al., 1994) (**Table 1**).

**Table 1:** Participant information.

	AN		HC		
	*M*	SD	*M*	SD	*p*
Age	22.18	5.45	22.64	3.25	0.725
Premorbid IQ	104.22	8.07	105.71	7.13	0.505
BMI	16.47	1.13	22.36	3.66	0.001
Illness duration	6.89	7.28	–	–	–
Age of illness onset	15.74	3.24	–	–	–
EDE-Q restraint	3.84	1.38	0.58	0.64	0.001
EDE-Q eating concern	3.79	1.27	0.25	0.32	0.001
EDE-Q shape concern	5.02	0.92	1.15	0.86	0.001
EDE-Q weight concern	4.50	1.45	0.60	0.77	0.001
EDE-Q global score	4.29	1.15	0.65	0.54	0.001
TAS-20 difficulty	22.78	5.88	11.46	4.74	0.001
identifying feelings					
TAS-20 difficulty	17.96	3.36	10.50	4.08	0.001
describing feelings					
TAS-20 externally	20.39	3.39	17.33	5.29	0.023
oriented thinking					
TAS-20 score	61.13	9.06	38.13	11.41	0.001

*AN, anorexia nervosa; HC, healthy control; Premorbid IQ, Standardized Wechsler Test of Adult Reading Score; BMI, body mass index; EDE-Q, Eating Disorders Examination Questionnaire; TAS-20, Toronto Alexithymia Scale; Age, age of illness onset and duration illness are reported in years.*

### Task

Participants were presented with face stimuli from a standard set of black-and-white images, the Pictures of Facial Affect (Ekman et al., 1975). The stimuli consist of male and female images displaying the seven basic emotions: anger, disgust, fear, happiness, sadness, surprise, and neutral. The stimuli chosen were those with the highest inter-rater agreement for all seven emotions. Four male and four female models displaying each emotion were presented. Participants were first presented with an implicit task while undergoing fMRI and eyetracking, followed by the explicit task which involved emotion identification and eyetracking. An implicit task was undertaken during fMRI as explicit tasks have a higher cognitive demand and more frontal areas involvement, making it more difficult to observe activity in areas involved in emotion processing (Critchley et al., 2000; Gorno-Tempini et al., 2001). In the implicit task, participants were presented with each stimulus twice in a pseudorandom fashion, resulting in a total of 16 presentations of each emotion over two runs. In the explicit task, participants were shown each stimulus once, resulting in eight presentations per emotion over one run. Due to the extended length of the task in the MRI, participants were not presented with surprised faces in the implicit task as it is the most ambiguous emotion, having neither a positive or negative emotional valence. Participants were also pseudorandomly presented with a black-and-white image of their own face with a neutral expression, with 16 presentations during the implicit task and eight during the explicit task. Photographs of participants were taken by the researcher while participants were instructed to look straight ahead with a relaxed expression, similarly to a passport photograph. Participants’ own face photographs were edited to match the properties of the Ekman face stimuli in terms of size and resolution, and were made black-and-white.

In the implicit task, faces were displayed pseudorandomly across emotions for 8000 ms on a white background, followed by a black 1° fixation cross for 3000–4300 ms. Each photograph was 336 × 640 pixels, equalling 17 cm × 27.5 cm or 18 × 13° at the eye. Participants were instructed simply to look at the face while it was on screen and to make a gender response with their right hand by clicking one of two buttons only when the fixation cross appeared following the face presentation. Long periods of fixation, between 10200 and 11400 ms were presented pseudorandomly throughout the task to increase BOLD signal variance by allowing the signal to return to baseline. Each of the two runs also began with a long period of fixation for 15000 ms.

In the explicit task, faces were again displayed for 8000 ms on a white background, equalling to 8 × 13° to the eye. Prior to the presentation of each stimulus, a 1° fixation cross appeared in the center of the screen. Following the presentation of each face stimulus, a forced-choice screen appeared on the monitor asking participants to identify the emotion displayed in the previous face from a list containing all of the emotions. The participants were given as much time as they required to make a response.

### Data Acquisition and Analysis

#### Eyetracking

Stimuli were presented through SR Research’s Experiment Builder program, and eyetracking was recorded using a remote view eyetracker, the EyeLink1000 (SR Research, Mississauga, ON, Canada), monocularly at 500 Hz. The recorded data were analyzed with SR Research’s analysis program, DataViewer. Areas of interest (AOIs) were defined as the eyes, nose and mouth. To investigate the proportion of fixations and fixation durations to salient features and non-salient features, two spatial-temporal parameters were calculated: the feature fixation index (FFI) and the feature duration index (FDI; Williams et al., 1999). The FFI is derived by dividing the number of fixations to salient features minus the number of fixations to non-salient features by the total number of fixations. The FDI is derived in the same manner. Indices range from -1 to +1, with positive values indicating more fixations or longer durations to salient features, and negative values indicating more visual attention to non-salient features.

#### Functional Magnetic Resonance Imaging

Magnetic resonance imaging scans were undertaken with the Siemens Tim Trio 3 tesla system with a 32 channel head coil at Swinburne University of Technology (Melbourne, VIC, Australia). During each functional run of active task performance, 1080 T2^∗^-weighted images were acquired axially parallel to the AC–PC line using an interleaved multiband sequence (multiband acceleration factor = 4, bandwidth = 25988 Hz/Px, repetition time (TR) = 710 ms, echo-time (TE) = 30 ms, echo-spacing = 0.51 ms, flip-angle = 52°, field of view = 222 mm, voxel resolution = 3 mm × 3 mm × 3 mm, slice thickness = 3 mm, number of slices = 44). Multiband acquisition sequences were derived from the Human Connectome Project (Moeller et al., 2010). A T1-weighted image was acquired sagitally for anatomical reference (bandwidth = 170 Hz/Px, TR = 1900 ms, TE = 2.52 ms, echo spacing = 7.5 ms, flip angle = 9°, field-of-view = 350 mm × 263 mm × 350 mm, voxel resolution = 1 mm × 1 mm × 1 mm, slice thickness = 1 mm).

Magnetic resonance imaging data pre-processing and statistical analyses were performed using SPM8, through Matlab R2014a (Mathworks, Natick, MA, USA). Image pre-processing included image realignment, then coregistration of the T1 image to a mean realigned functional image created during realignment. The co-registered T1 image was normalized to the T1 template supplied with SPM8 Montreal Neuroimaging Institute (MNI), then the parameters of this transformation were applied to realigned functional images. The normalized functional images were spatially smoothed with a Gaussian kernel of 8 mm × 8 mm × 8 mm.

#### Statistical Analyses

First-level modeling was performed by fitting a convolved hemodynamic response function (HRF) and its temporal derivative separately to the onset times of angry, disgusted, fearful, happy, sad, neutral, and own faces (seven regressors plus their temporal derivative). After parameter estimation, each emotion parameter and the participants’ own face parameter was contrasted with the neutral face parameter producing six contrast images (angry > neutral, sad > neutral, etc.). At the group level, these contrast images were first entered into one-way analysis of variance (ANOVA) models for AN and HC groups separately to investigate within-group effects (results are presented in Supplementary Material). Group differences were interrogated with a mixed-effects ANOVA model using the flexible factorial option in SPM8. This model included a between-subjects *group* factor (two levels: patients vs. controls), a within subjects *condition* factor (six levels: angry > neutral, sad > neutral, etc.) and a *subject*s factor (number of levels equals the number of participants) that controlled for within-subject variability (Gläscher and Gitelman, 2008).

*T*-statistic images were corrected for multiple comparisons using the random-field theory approach at the voxel and cluster levels (*p* < 0.05, FWE-corrected). The mixed-design analysis involved the investigation of a group by condition interaction, followed by simple effects comparing each condition between groups.

Following the mixed-design analysis, clusters which resulted in significant differences between groups were defined as different regions of interests (ROIs) with the MarsBar toolbox (Brett et al., 2002) run under Matlab R2014a. Contrast estimates for each ROI were correlated with eyetracking and behavioral data, and scores on the EDE-Q and TAS-20.

Performance on eyetracking components and emotion identification (rate of emotion identification errors) were compared with mixed design ANOVAs, following normality checking and the removal of outliers. Percentage data underwent an arcsine transformation prior to inclusion in ANOVAs. Violations of sphericity were corrected with a Greenhouse–Geiser correction. For conditions in which groups significantly differed in emotion identification error rate, Mann–Whitney *U* tests were carried out to identify which emotions were incorrectly reported as the data were not normally distributed. Pearson’s correlation analyses were also performed between eyetracking data and behavioral data, and scores on the EDE-Q and TAS-20. For brevity, only significant interactions with group will be commented on in detail. Detailed results of eyetracking and fMRI analyses unrelated to group interactions are available as Supplementary Material.

## Results

### Behavioral

For rate of emotion identification errors, a 2 (group) × 8 (condition) mixed design ANOVA was undertaken (see Supplementary Table S1). The analysis revealed a significant main effect of condition [*F*(2.5,113.5) = 7.4, *p* < 0.001]. A significant main effect of group [*F*(1,46) = 5.2, *p* ≤ 0.05] and a significant interaction between group and condition were also found [*F*(2.5,113.5) = 4.6, *p* ≤ 0.01]. Within subjects contrasts revealed that groups did not significantly differ in emotion identification errors to any individual emotion, but AN participants made significantly more emotion identification errors to their own face [*F*(1,46) = 5.1, *p* ≤ 0.05]. When errors were made to own face emotion, analyses revealed that AN participants were more likely than controls to report their own face as sad [*U*(46) = 200.0, Z = -2.9, *p* ≤ 0.01], whereas control participants were more likely to correctly report their own neutral face as portraying a neutral expression [*U*(46) = 209.0, *Z* = -2.3, *p* ≤ 0.05] (see Supplementary Table S2).

### Eyetracking

Average fixation count, fixation duration, and saccade amplitude were analyzed in separate 2 (group) × 7 (condition) × 2 (task) mixed design ANOVAs. As the first run of the fMRI task consisted of the same number of trials as the behavioral task, these two tasks were included in the analysis. Furthermore, as the behavioral task also consisted of surprised faces which were not included in the fMRI task, these trials were excluded from the analysis. Means and SD for fixation count, fixation duration and saccade amplitude are presented in see Supplementary Table S3.

For fixation count, a significant main effect of condition [*F*(4.1,159.2) = 3.0, *p* ≤ 0.05] was found with a greater number of fixations made to participants’ own faces and faces depicting anger and fear. A significant main effect was also found for group [*F*(1,39) = 5.3, *p* ≤ 0.05], with AN participants making more fixations than controls. A significant interaction between condition and task was also found [*F*(3.4,131.9) = 7.61, *p* < 0.001] with a decreased number of fixations between implicit and explicit tasks for participants’ own faces and faces depicting fear. There was, however, no significant main effect of task, and no significant interaction between condition and group, or task and group. There was also no interaction between condition, group and task. Analyses conducted on fixation duration revealed a significant main effect of group [*F*(1,38) = 8.5, *p* ≤ 0.01] with AN participants making fixations of shorter duration than heathy individuals. No other significant main effects or interactions were found. Analyses conducted on saccade amplitude resulted in no significant main effects or interactions.

A 2 (group) × 7 (condition) × 2 (task) mixed design ANOVA conducted on the FDI revealed a significant main effect of condition [*F*(2.3,82.0) = 16.4, *p* < 0.001], and a significant interaction between condition and task [*F*(4.0,144.9) = 3.0, *p* ≤ 0.05] with greater attention to salient features of one’s own face during the implicit compared to explicit task. Analyses conducted on the FFI revealed significant main effects of condition [*F*(2.2,72.5) = 19.7, *p* < 0.001] and task [*F*(1,33) = 10.1, *p* ≤ 0.01] with greater attention to salient facial features during the explicit task compared to the implicit task. Significant interactions were also found between condition and task [*F*(3.8,125.9) = 3.1, *p* ≤ 0.05] and condition and group [*F*(2.2,72.5) = 3.2, *p* ≤ 0.05]. Within-subjects contrasts did not reveal any significant differences between groups for any emotion, but a significant difference for own faces between AN and control participants [*F*(1,33) = 5.9, *p* ≤ 0.05]. Further 2 (group) × 2 (task) mixed design ANOVAs were also conducted on the FFI and FDI to participants’ own face. A significant main effect of task, and an interaction between task and group were not found for either the FFI or FDI. A significant main effect of group was, however, found for both the FFI [*F*(1,36) = 7.6, *p* > 0.01] and FDI [*F*(1,36) = 6.8, *p* ≤ 0.05] (see Supplementary Table S4). Control participants showed more visual attention to salient features of their own face, whereas the attention shown to salient and non-salient features of their own face in AN was roughly equal.

### Functional Magnetic Resonance Imaging

#### Mixed Design Analysis

**FIGURE 1:**
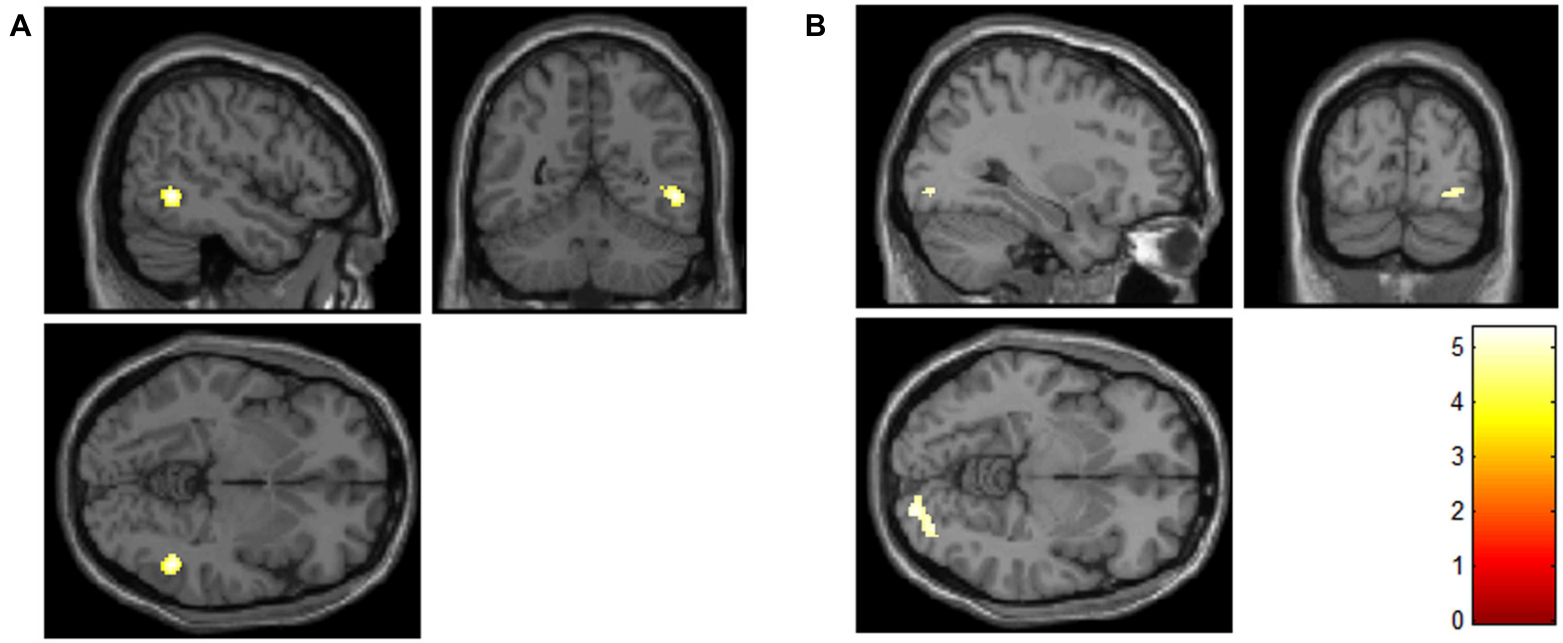
**Increased activity in the anorexia nervosa (AN) group compared to the control group in the right inferior and middle temporal gyri **(A)**, and the right lingual gyrus **(B)** for participants’ own faces compared to neutral faces (contrast: AN > controls, own > neutral face; FWE-corrected for multiple comparisons at the voxel and cluster levels).** The color scale indicates the *t*-value.

The analysis did not result in a significant group by condition interaction. Simple effects between groups for each condition revealed a significant difference between groups only for the own > neutral face contrast. Increased activation was found in AN compared to controls in the own > neutral contrast in two clusters: one in the right inferior temporal and middle temporal gyri, and one in the right lingual gyrus (**Table 2**; **Figure 1**).

**Table 2:** Significant activations for participants’ own faces compared to neutral faces between anorexia nervosa (AN) and control participants, Own > Neutral, AN > Healthy Controls.

Peak regions	No. of voxels	Peak *t*	Peak MNI coordinates		
			*x*	*y*	*z*
Inferior temporal gyrus	104	6.45	50	-54	-4
middle temporal gyrus					
Lingual gyrus	118	5.37	28	-88	-8

*MNI, Montreal Neuroimaging Institute.*

### Pearson’s Correlations

BOLD activity in the own > neutral face contrast in either the inferior and middle temporal gyri ROI, or the lingual gyrus ROI did not significantly correlate with any eyetracking parameter, rate of emotion identification errors, or the results of the EDE-Q and TAS-20 for either group. Eyetracking and behavioral data, and scores on the EDE-Q and TAS-20 were also not found to correlate with one another.

## Discussion

The aim of this study was to investigate own face processing and the processing of emotional faces of others, in individuals with AN. Although the AN group were found to have higher alexithymia scores, they did not differ from healthy controls in emotion identification of the Ekman emotional face stimuli. Groups were found to differ in visual scanpath behavior to the stimuli in general, with AN participants demonstrating hyperscanning, evinced by increased fixations of shorter duration, relative to controls. AN participants were also found to avoid visually attending to salient features of their own face and displayed increased activity in the right lingual, and inferior and middle temporal gyri to images of their own face, compared to neutral control images, as well as to healthy individuals.

In analyses directly comparing AN to healthy individuals, AN participants showed increased activity to their own face in the right inferior and middle temporal gyri, and lingual gyrus, areas related to higher-order visual perception. Increased activity of the lingual gyrus has been reported during the processing of human faces (Kesler/West et al., 2001), and increased activity in the inferior and middle temporal gyri have been specifically found in response to own face stimuli (Kircher et al., 2001; Platek et al., 2006; Sugiura et al., 2008). Therefore, the findings of the current study suggest an increased processing of own face stimuli in AN in areas related to visual perception of self.

Differences in lingual gyrus and temporal gyrus activity have also been reported when individuals with AN are presented with images of their own body compared to other individuals’ bodies. However, increased activity in these areas was found for controls relative to AN participants (Sachdev et al., 2008; Vocks et al., 2010), rather than increased activity in AN as found in the current study. However, neither of those studies actually involved face processing; rather, the faces of the stimuli presented in those studies were masked when presented to participants. Therefore, the differences in activation between the current findings and the findings of these two studies may be related to the specific processing of self-face and self-body images which result in increased activity and decreased activity in these areas in AN respectively.

Our AN group also showed more visual attention to non-salient features and avoided fixating on salient features of their own face. Freeman et al. (1991) reported that when AN participants were presented with whole body images of themselves, less visual attention was allocated to their faces compared to controls, though the level of visual attention to different body areas did not differ. Furthermore, Giel et al. (2011) reported a similar pattern of visual attention in a study which presented AN and control participants with food and non-food images simultaneously: although healthy individuals showed more visual attention to food stimuli, the AN group displayed roughly equal attention to food and non-food stimuli. These results may be related to the finding that decreased visual attention is associated with the presentation of anxiety-inducing and phobic stimuli. Similarly to the current findings, Horley et al. (2003) reported that individuals with social anxiety disorder made fixations of short duration to salient features of emotional faces stimuli. Pflugshaupt et al. (2007), reported that individuals with spider phobia displayed fewer fixations to spider images and instead diverted their attention to neutral areas of the stimulus. The authors described this behavior as a strategy to cope with threatening and confrontational stimuli, to consequently reduce anxiety. Individuals with AN may utilize similar strategies when viewing their own face as they may find these stimuli anxiety-provoking. An avoidance of salient features was not, however, found to the Ekman face stimuli, suggesting that this behavior is specific to one’s own face.

Hyperscanning of face stimuli (increased fixations of shorter duration) was also found in our AN group, though this was not specific to self-face images. Hyperscanning behaviors are associated with increased anxiety, as has been reported in social anxiety disorder to face stimuli of different emotions (Horley et al., 2003). Horley et al. (2003) suggested the hyperscanning behavior in social anxiety disorder may reflect a fear of social evaluation. This explanation may also be relevant to the current findings: due to a preoccupation with physical appearance, individuals with AN are particularly sensitive to social evaluations made by others (Striegel-Moore et al., 1993) and may show increased scanning behaviors to related stimuli.

Despite previous reports of emotion identification deficits in individuals with AN, our findings do not concur. Although higher levels of alexithymia were found in the AN group, the ability to identify emotions from a standard set of face stimuli did not differ from healthy individuals. This finding is consistent with the literature which suggests that alexithymia levels are not related to the ability to perceive emotion from faces (see Grynbergs et al., 2012 for a review). However, whether high alexithymia impairs the ability to perceive emotion in an image of one’s own face has not been investigated, nor was it specifically investigated in this study, as we only presented participants with own-face images showing a neutral expression. Although the majority of AN participants reported their own face as portraying a neutral expression, they were more likely than controls to report their own face as depicting a sad expression. However, rather than indicating an emotion identification deficit specific to themselves, this result is more likely to reflect a biased self-perception in how individuals with AN *feel* they look. Thus, when identification errors occurred as they viewed their own faces, they were specific to seeing themselves as sad and not any other emotions. Furthermore, when participants were questioned following the task about their overall experience, the controls often reported that they found it ‘funny’ looking at their own face, whereas AN patients tended to feel disgusted.

Though AN and control participants differed in the processing of their own face, we found no differences in BOLD activity in response to different emotions, in either group. As strict thresholding was utilized in an attempt to correct for multiple comparisons, the task may not have had sufficient power to document significant differences in activation of each emotion relative to the neutral face condition. However, as the contrasts between participants’ own face and the neutral face condition survived this threshold, the results show that BOLD activity elicited in response to the different emotions was not as strong. Since we were interested in assessing the visual scanpaths of AN patients to face stimuli, the extended presentation time was required for this purpose and the number of trials was therefore limited to remain within a reasonable duration for an MRI task. In future research, where visual scanpaths are not of interest, an increased number of trials should be utilized. It would also be of interest to investigate differences in visual scanpaths and neural activity to both own-face and own-body stimuli in AN, and an evaluation of how these images make AN patients feel.

The findings of this study have important potential clinical implications. Participants with AN did not differ from healthy control participants in the areas of attentional focus when viewing the Ekman face stimuli, nor did they differ in emotion identification of these stimuli. AN participants did, however, display an avoidance of salient features of images of their own faces. This may have consequently led to the mislabelling of their own neutral expression as sad. In other words, the perception of one’s own face with a neutral expression as appearing sad in AN may be related to patients not looking at the correct areas of their own face when making an affect judgment. This may be as a result of emotional disturbances and alexithymia in AN, or this alternatively may lead to these disturbances. Therefore, remediation techniques which train participants to focus on the correct areas of the face may be beneficial in AN. These techniques have proven useful in other psychiatric conditions such as schizophrenia, with trained individuals demonstrating an improvement in attention to salient facial features and emotion recognition (Russell et al., 2008; Marsh et al., 2012). Furthermore, the majority of deficits reported in the current study were specific to the processing of self-images in AN. This emphasizes the importance of therapies such as cognitive behavioral therapy to address distorted perceptions of oneself in AN and correctly analyzing events and the patient’s own internal dialog. It is also possible that the sense of disgust evoked by viewing their own face also reduced the AN group’s fixations on the salient regions of images of their own faces, in the way that one might avoid eye contact with a repellent individual.

In summary, this study suggests intact emotion identification of facial affect stimuli and distinct hyperscanning behaviors when viewing faces in AN. Significant differences were also found in the processing of one’s own face in AN, with AN participants showing a greater level of visual attention to non-salient features and increased activity in inferior and middle temporal, and lingual gyri, relative to healthy individuals. These findings suggest overlap with anxiety disorders, as evinced by the hyperscanning behaviors displayed, and increased anxiety, particularly to own face images evinced by an apparent avoidance of salient features. Together with the fMRI finding, the study suggests that the processing of self-face images is different in AN, and may contribute to the distorted perception of oneself experienced by individuals with this illness.

## Author Contributions

AP, LA, SR, CG, and DC designed the research; AP completed data collection; all authors were involved in data analysis and manuscript preparation.

## Conflict of Interest Statement

Prof. David J. Castle reports grants and personal fees from Eli Lilly, grants and personal fees from Janssen-Cilag, grants and personal fees from Roche, grants and personal fees from Allergen, grants and personal fees from Bristol-Myer Squibb, grants and personal fees from Pfizer, grants and personal fees from Lundbeck, grants and personal fees from AstraZeneca, grants and personal fees from Hospira, during the conduct of the study; personal fees from Eli Lilly, personal fees from Bristol-Myer Squibb, personal fees from Lundbeck, personal fees from Janssen-Cilag, personal fees from Pfizer, personal fees from Organon, personal fees from Sanofi-Aventis, personal fees from Wyeth, personal fees from Hospira, personal fees from Servier, outside the submitted work. Prof. Larry A. Abel reports personal fees from Actelion Pharmaceuticals, Switzerland, outside the submitted work. Dr. Andrea Phillipou, Prof. Susan L. Rossell, Dr. Caroline Gurvich, Dr. Matthew E. Hughes, and Mr. Richard G. Nibbs report no conflicts of interest.
