# Supplementary material for: Self perception and facial emotion perception of others in anorexia nervosa
Source: Front Psychol. 2015 Aug 10;6:1181. doi: 10.3389/fpsyg.2015.01181 (PMC4530666; doi:10.3389/fpsyg.2015.01181)
Supplement: Supplementary file 1 [file Data_Sheet_1.DOCX]

***Supplementary Material***

**Self perception and facial emotion perception of others in anorexia nervosa**

Andrea Phillipou*^a,b,c^, Larry Allen Abel^a^, David Jonathan Castle^d,b,e^, Matthew Edward Hughes^f^, Caroline Gurvich^g^, Richard Grant Nibbs^f^ and Susan Lee Rossell^f,g,d^.

^a^ Department of Optometry & Vision Sciences, The University of Melbourne, Melbourne, VIC, Australia

^b^ Department of Psychiatry, The University of Melbourne, Melbourne, VIC, Australia

^c^ Department of Mental Health, The Austin Hospital, Melbourne, VIC, Australia

^d^ Department of Psychiatry, St Vincent’s Hospital, Melbourne, VIC, Australia

^e^ Faculty of Health Sciences, Australian Catholic University, Melbourne, VIC, Australia

^f^ Brain and Psychological Sciences Research Centre, Swinburne University of Technology, Melbourne, VIC, Australia

^g^ Monash Alfred Psychiatry Research Centre, Melbourne, VIC, Australia

*Correspondence:

Dr Andrea Phillipou

Department of Mental Health

St Vincent’s Hospital

Fitzroy, VIC, 3065

Australia

[ap@unimelb.edu.au](mailto:ap@unimelb.edu.au)

Behavioural results

Table S1: Rate of emotion identification errors

|  | AN | | HC | |  |  |  |
| --- | --- | --- | --- | --- | --- | --- | --- |
|  | M | SD | M | SD | F | *p* | Cohen’s d |
| Anger | 16.30 | 11.58 | 22.00 | 20.18 | 1.41 | 0.242 | 0.35 |
| Disgust | 9.78 | 14.58 | 12.50 | 16.14 | 0.37 | 0.545 | 0.18 |
| Fear | 22.28 | 19.20 | 17.00 | 16.09 | 1.07 | 0.306 | 0.30 |
| Happy | 2.17 | 6.14 | 1.50 | 4.15 | 0.20 | 0.656 | 0.13 |
| Sad | 21.74 | 16.52 | 13.50 | 15.28 | 3.22 | 0.079 | 0.52 |
| Surprise | 8.70 | 9.56 | 4.50 | 7.97 | 2.74 | 0.104 | 0.48 |
| Neutral | 9.78 | 23.52 | 2.50 | 5.10 | 2.28 | 0.138 | 0.43 |
| Own | 32.61 | 44.07 | 5.50 | 20.44 | 7.68 | 0.008 | 0.79 |

Note: AN= anorexia nervosa; HC= healthy control; rate of errors are reported as percentages

Table S2: Own face emotion responses, behavioural task.

|  | AN | |  | HC | |  |  |  |
| --- | --- | --- | --- | --- | --- | --- | --- | --- |
|  | Median | Range |  | Median | Range | U | z | *p* |
| Anger | 0.00 | 0.00 |  | 0.00 | 0.00 | 287.50 | 0.00 | 1.000 |
| Disgust | 0.00 | 100.00 |  | 0.00 | 0.00 | 262.50 | -1.49 | 0.136 |
| Fear | 0.00 | 12.50 |  | 0.00 | 0.00 | 275.00 | -1.04 | 0.297 |
| Happy | 0.00 | 0.00 |  | 0.00 | 100.00 | 264.50 | -1.37 | 0.170 |
| Sad | 0.00 | 100.00 |  | 0.00 | 0.00 | 200.00 | -2.94 | 0.003 |
| Surprise | 0.00 | 12.50 |  | 0.00 | 0.00 | 275.00 | -1.04 | 0.297 |
| Neutral | 100.00 | 100.00 |  | 100.00 | 100.00 | 209.00 | -2.29 | 0.022 |

Note: AN=Anorexia Nervosa; HC=healthy control; rates are reported as percentages

Eyetracking results

For fixation count, a 2 (group) x 7 (condition) x 2 (task) mixed design ANOVA revealed a significant main effect of condition (F(4.1,159.2)= 3.0, p<.05) with a greater number of fixations made to participants’ own faces (F(1,39)= 5.3, p<.05), and faces depicting anger (F(1,39)= 5.4, p<.05) and fear (F(1,39)= 11.0, p<.01). A significant interaction between condition and task was also found (F(3.4,131.9)= 7.6, p < 0.001) with a decreased number of fixations between implicit and explicit tasks for participants’ own faces (F(1,39)=15.9, p < 0.001) and faces depicting fear (F(1,39)= 13.5, p< 0.001).

A 2 (group) x 7 (condition) x 2 (task) mixed design ANOVA conducted on the FDI revealed a significant main effect of condition (F(2.28,81.95)= 16.37, p < 0.001) with longer fixations to salient features of own faces (F(1,36)= 5.95, p= 0.020), and faces depicting anger (F(1,36)= 12.34, p= 0.001), disgust (F(1,36)= 24.77, p < 0.001), fear (F(1,36)= 42.40, p < 0.001), happiness (F(1,36)= 30.53, p < 0.001) and sadness (F(1,36)= 29.19, p < 0.001). A significant interaction between condition and task was also found with greater attention to salient features of one’s own face during the implicit compared to explicit task (F(1,36)= 5.39, p= 0.026).

A 2 (group) x 7 (condition) x 2 (task) mixed design ANOVA conducted on the FFI revealed a significant main effect of condition with a greater number of fixations to salient features of own faces (F(1,33)= 9.61, p= 0.004), and faces depicting anger (F(1,33)= 7.38, p= 0.010), disgust (F(1,33)= 23.38, p < 0.001), fear (F(1,33)= 58.26, p < 0.001), happiness (F(1,33)= 24.32, p < 0.001), and sadness (F(1,33)= 32.66, p < 0.001). A significant interaction between condition and task was also found, with more attention to salient features of own face stimuli during implicit compared to explicit tasks (F(1,33)= 4.56, p= 0.040).

Table S3: Scanpath characteristics to faces of different emotion during the fMRI and behavioural tasks

|  | Fixation Count | | | | Fixation duration | | | | Saccade Amplitude | | | |
| --- | --- | --- | --- | --- | --- | --- | --- | --- | --- | --- | --- | --- |
|  | AN | | HC | | AN | | HC | | AN | | HC | |
|  | M | SD | M | SD | M | SD | M | SD | M | SD | M | SD |
| *fMRI task part 1* |  |  |  |  |  |  |  |  |  |  |  |  |
| Anger | 23.97 | 10.90 | 20.04 | 3.87 | 282.95 | 109.63 | 336.81 | 103.66 | 1.89 | 0.56 | 1.99 | 0.35 |
| Disgust | 23.86 | 9.88 | 19.77 | 4.55 | 287.10 | 114.91 | 408.71 | 220.44 | 1.72 | 0.43 | 1.91 | 0.36 |
| Fear | 24.60 | 11.25 | 20.37 | 4.15 | 268.07 | 88.64 | 382.34 | 157.42 | 1.82 | 0.42 | 1.99 | 0.35 |
| Happy | 24.74 | 9.25 | 20.27 | 3.57 | 274.46 | 92.20 | 401.93 | 192.99 | 1.77 | 0.51 | 1.97 | 0.43 |
| Sad | 23.56 | 10.48 | 20.09 | 3.66 | 295.76 | 121.29 | 334.28 | 105.24 | 1.86 | 0.52 | 1.89 | 0.41 |
| Neutral | 23.01 | 10.43 | 19.30 | 3.78 | 291.72 | 126.37 | 356.55 | 136.97 | 1.89 | 0.56 | 2.03 | 0.39 |
| Own | 25.26 | 11.03 | 21.58 | 4.57 | 281.94 | 102.45 | 369.41 | 152.33 | 1.87 | 0.50 | 1.89 | 0.43 |
| *Behavioural task* |  |  |  |  |  |  |  |  |  |  |  |  |
| Anger | 23.75 | 7.29 | 21.53 | 3.80 | 291.97 | 144.74 | 313.07 | 55.73 | 2.12 | 0.49 | 1.97 | 0.26 |
|  |  |  |  |  |  |  |  |  |  |  |  |  |
| *Table S3 cont’d* |  |  |  |  |  |  |  |  |  |  |  |  |
| Disgust | 23.04 | 6.17 | 20.79 | 4.61 | 283.36 | 101.50 | 321.48 | 60.73 | 2.10 | 0.54 | 1.89 | 0.37 |
| Fear | 24.53 | 7.42 | 21.84 | 4.45 | 264.58 | 78.91 | 321.13 | 84.92 | 2.14 | 0.50 | 1.99 | 0.28 |
| Happy | 22.02 | 7.09 | 19.89 | 4.28 | 274.09 | 93.37 | 334.63 | 84.73 | 2.23 | 0.73 | 2.04 | 0.39 |
| Sad | 23.74 | 6.71 | 21.07 | 3.84 | 268.48 | 79.34 | 323.16 | 59.44 | 2.02 | 0.58 | 1.89 | 0.34 |
| Neutral | 23.53 | 7.05 | 20.19 | 4.09 | 253.98 | 73.94 | 348.81 | 118.21 | 2.21 | 0.74 | 2.08 | 0.47 |
| Own | 21.82 | 8.12 | 19.17 | 2.97 | 288.34 | 77.60 | 370.75 | 90.52 | 2.34 | 1.19 | 2.05 | 0.41 |

Note: AN=anorexia nervosa; HC=healthy control; fixation duration is reported in milliseconds and saccade amplitude in degrees

Table S4: Feature fixation index and feature duration index scores for faces of different emotion during the fMRI and behavioural tasks

|  | Feature fixation index | | | | Feature duration index | | |  |
| --- | --- | --- | --- | --- | --- | --- | --- | --- |
|  | AN | | HC | | AN | | HC | |
|  | M | SD | M | SD | M | SD | M | SD |
| *fMRI task part 1* |  |  |  |  |  |  |  |  |
| Anger | 0.16 | 0.33 | 0.15 | 0.37 | 0.25 | 0.26 | 0.27 | 0.33 |
| Disgust | 0.23 | 0.36 | 0.20 | 0.38 | 0.34 | 0.26 | 0.34 | 0.34 |
| Fear | 0.24 | 0.32 | 0.23 | 0.37 | 0.38 | 0.24 | 0.31 | 0.33 |
| Happy | 0.26 | 0.33 | 0.24 | 0.41 | 0.36 | 0.23 | 0.36 | 0.38 |
| Sad | 0.17 | 0.36 | 0.17 | 0.37 | 0.28 | 0.25 | 0.31 | 0.33 |
| Neutral | 0.14 | 0.36 | 0.11 | 0.39 | 0.20 | 0.29 | 0.25 | 0.39 |
| Own | -0.06 | 0.40 | 0.17 | 0.30 | 0.08 | 0.34 | 0.28 | 0.29 |
| *Behavioural task* |  |  |  |  |  |  |  |  |
| Anger | 0.40 | 0.30 | 0.41 | 0.23 | 0.36 | 0.41 | 0.46 | 0.25 |
| Disgust | 0.39 | 0.29 | 0.45 | 0.25 | 0.32 | 0.47 | 0.49 | 0.29 |
| Fear | 0.5 | 0.24 | 0.50 | 0.25 | 0.41 | 0.43 | 0.52 | 0.31 |
| Happy | 0.40 | 0.29 | 0.41 | 0.15 | 0.39 | 0.42 | 0.46 | 0.27 |
| Sad | 0.45 | 0.28 | 0.45 | 0.22 | 0.40 | 0.41 | 0.51 | 0.26 |
| Neutral | 0.36 | 0.26 | 0.33 | 0.24 | 0.30 | 0.40 | 0.36 | 0.31 |
| Own | 0.08 | 0.33 | 0.22 | 0.28 | 0.06 | 0.43 | 0.25 | 0.34 |

Note: AN=anorexia nervosa; HC=healthy control

fMRI results

One-way within groups ANOVAs

*AN*

For the AN group, a significant main effect of condition was found. Simple effects are presented in Table S5. The own > neutral contrast was the only contrast to result in significant activations at this threshold, revealing widespread activation in bilateral areas including clusters within the inferior frontal, temporal and occipital cortices. Significant activations were also revealed within the right supplementary motor area, and anterior and mid cingulate cortices. Significant activations within the left hemisphere included clusters within the precentral sulcus, superior and inferior parietal cortices, and superior and medial frontal areas.

*HC*

A significant main effect of condition was also found for the control group. Individual contrasts for each emotion compared to neutral did not result in any significant activations at this threshold. Significant activations were found in the own face > neutral face comparison. Similarly to the finding in the AN group, widespread activation was found to control participants’ own face in comparison to the neutral affect faces (Table S6), but unlike the bilateral pattern observed with AN participants, the activations observed in the control group were predominantly located in the right hemisphere. Activations in the right hemisphere included clusters within the superior parietal and occipital cortices, inferior and mid frontal cortices, inferior parietal, temporal and occipital cortices, mid cingulate cortex, precuneus, supplementary motor area, precentral sulcus, and the insula. Significant activations within the left hemisphere included clusters in inferior and superior frontal areas, the insula and precuneus.

Table S5: Significant within groups contrasts for the Emotional Faces task for Anorexia Nervosa participants, Own>Neutral

| Peak regions | No. of voxels | Peak t | Peak MNI coordinates | | |
| --- | --- | --- | --- | --- | --- |
|  |  |  | x | y | z |
| Inferior frontal | 4880 | 12.27 | 46 | 0 | 32 |
| Brainstem, thalamus | 1012 | 10.22 | 4 | -28 | -6 |
| Inferior occipital, inferior temporal | 4267 | 9.92 | 42 | -82 | 16 |
| Superior parietal | 2667 | 8.77 | -16 | -72 | 48 |
| Inferior frontal, insula | 1920 | 8.73 | -34 | 24 | -6 |
| Precentral sulcus | 717 | 7.55 | -46 | -2 | 36 |
| Inferior occipital, inferior temporal | 445 | 7.84 | -44 | -68 | -6 |
| Superior frontal | 119 | 7.34 | -8 | 50 | 54 |
| Supplementary motor | 1163 | 7.09 | 8 | 16 | 68 |
| Anterior and mid cingulate | 621 | 6.19 | 2 | 22 | 20 |
| Medial frontal | 217 | 5.68 | -6 | 48 | 24 |
| Inferior parietal | 171 | 5.66 | -62 | -42 | 40 |

Note: MNI=Montreal Neuroimaging Institute

Table S6: Significant within groups contrasts for the Emotional Faces task for control participants, Own>Neutral.

| Peak regions | No. of voxels | Peak t | Peak MNI coordinates | | |
| --- | --- | --- | --- | --- | --- |
|  |  |  | x | y | z |
| Inferior and mid frontal, precentral sulcus, | 3318 | 11.85 | 48 | 2 | 30 |
| insula |  |  |  |  |  |
| Superior parietal, superior occipital, | 1686 | 9.58 | 22 | -66 | 48 |
| precuneus |  |  |  |  |  |
| Supplementary motor area | 517 | 7.87 | 8 | 6 | 64 |
| Inferior frontal, insula | 991 | 7.78 | -36 | 22 | -4 |
| Inferior temporal, inferior occipital | 228 | 7.54 | 46 | -68 | -10 |
| Superior frontal | 303 | 6.49 | -8 | 52 | 44 |
| Mid cingulate | 146 | 6.45 | 8 | -6 | 32 |
| Inferior parietal | 133 | 6.34 | 56 | -34 | 44 |
| Mid cingulate | 189 | 6.29 | 6 | 24 | 38 |
| Precuneus | 108 | 6.08 | -10 | -68 | 34 |

Note: MNI=Montreal Neuroimaging Institute
